# Evaluating the Association between Contrast Medium Dosage and Acute Kidney Injury in Transcatheter Aortic Valve Replacement Using Different Predictive Models

**DOI:** 10.3390/jcm9113476

**Published:** 2020-10-28

**Authors:** Markus Mach, Waseem Hasan, Martin Andreas, Bernhard Winkler, Gabriel Weiss, Christopher Adlbrecht, Georg Delle-Karth, Martin Grabenwöger

**Affiliations:** 1 Department of Cardiac Surgery, Medical University of Vienna, 1090 Vienna, Austria; martin.andreas@meduniwien.ac.at; 2 Heart Team Vienna, Department of Cardio-Vascular Surgery, Vienna North Hospital—Clinic Floridsdorf and the Karl Landsteiner Institute for Cardio-Vascular Research, 1130 Vienna, Austria; winklermed@yahoo.com (B.W.); gabriel.weiss@me.com (G.W.); martin.grabenwoeger@wienkav.at (M.G.); 3 Faculty of Medicine, Imperial College London, London SW7 2AZ, UK; waseem.hasan15@imperial.ac.uk; 4 Vienna North Hospital—Clinic Floridsdorf and the Karl Landsteiner Institute for Cardiovascular and Critical Care Research, 1210 Vienna, Austria; c.adlbrecht@imed19.at (C.A.); georg.dellekarth@wienkav.at (G.D.-K.); 5 Medical Faculty, Sigmund Freud University, 1020 Vienna, Austria

**Keywords:** TAVR, AKI, mortality, renal function, contrast medium

## Abstract

Recent studies have suggested that contrast medium (CM) volume is associated with acute kidney injury (AKI) after transcatheter aortic valve replacement (TAVR). However, in a high-risk elderly TAVR population, the prognostic value and ideal threshold of CM dosage for AKI is unclear. Data of 532 successive TAVR patients (age 81.1 ± 6.8 years, EuroSCORE II 4.8% ± 6.0%) were therefore retrospectively analyzed. Based on a recently published formula, the renal function (preprocedural serum creatinine: SCr) corrected ratio of CM and body weight (CM*SCr/BW) was calculated to determine the risk of postprocedural contrast-associated AKI. AKI occurred in 94 patients (18.3%) and significantly increased 1-year all-cause mortality (23.4% vs. 13.1%; *p* = 0.001). A significant correlation between AKI and 30-day as well as 1-year all-cause mortality was observed (*p* = 0.001; *p* = 0.007). However, no association between CM dosage or the CM*SCr/BW ratio with the occurrence of AKI was seen (*p* = 0.968; *p* = 0.442). In our all-comers, all-access cohort, we found no relationship between CM dosage, or the established risk ratio model and the occurrence of postprocedural AKI. Further research needs to be directed towards different pathophysiological causes and preventive measures as AKI impairs short- and long-term survival.

## 1. Introduction

Acute kidney injury (AKI) is common in patients undergoing transcatheter aortic valve replacement (TAVR) and can lead to a substantial increase in mortality and hospitalization length [1,2,3,4].

The relationship between contrast medium (CM) application and renal impairment has been studied in percutaneous coronary intervention (PCI) [5,6,7], and set the groundwork for investigating AKI after TAVR by providing several models to draw from [6,8]. However, the prognostic value and threshold dosage of CM for AKI in TAVR remains uncertain. Conflicting results were published so far regarding the association of CM dosage and AKI in TAVR patients [1,8,9,10,11,12,13,14,15,16].

The possibility of CM dosage having a negligible effect on AKI development has precedent in related fields. No significant difference in AKI incidence between those who received CM and those who did not was found in patients with an estimated glomerular filtration rate (eGFR) greater than 30 mL/min/1.73 m2, undergoing computed tomography scanning [17,18,19]. Previous studies had already indicated these findings, with research groups suggesting that other factors including the underlying disease as the cause for AKI and nephropathy among patients receiving CM [20,21]. The latest consensus statement by the American College of Radiology also noted that risk of developing AKI after administering intravenous CM in renally impaired patients was overstated [22]. Given the different weighting that the risk of CM appears to have on AKI development depending on the procedure and route of administration, it is important to evaluate whether current practices regarding CM administration in TAVR are reasonable.

Despite efforts to reduce CM dosages in pre-TAVR computed tomography (CT) protocols [23], operators may ultimately still be faced with the decision whether to limit the administered CM volume (to prevent renal complications) at the expense of reduced imaging quality both pre- and periprocedurally. Determining whether CM dosage translates to meaningful changes in TAVR outcomes would help the clinical team prioritize the most important factors predisposing patients to AKI.

Our study therefore examined the relationship between CM volume and postprocedural AKI in the VIenna CardioThOracic Aortic Valve RegistrY (VICTORY) in order to determine cut-off values for CM dosages whilst simultaneously quantifying the effects of AKI on short- and long-term mortality.

## 2. Materials and Methods

A total of 532 patients from the VICTORY-Registry on whom a transapical (*n* = 266) or transfemoral TAVR (*n* = 266) was performed between June 2009 and December 2016 at the Heart Center Hietzing in Vienna (Austria) were investigated. TAVR procedures were performed when indicated by the institutional heart team in patients at high surgical risk. The parameters that defined the aforementioned risk were: a logistic European System for Cardiac Operative Risk Evaluation (Logistic EuroSCORE) >10% or, a Society of Thoracic Surgeons Predictive Risk of Mortality (STS) score or EuroSCORE II >4%.

A total of 514 patients were examined in the study as 18 patients had to be excluded due to their AKI incidence being invalid or immeasurable. The excluded population included 13 patients who died within 72 h of the TAVR, four who received regular hemodialysis prior to the procedure, and one patient that had been fast-tracked for discharge without post-interventional blood samples.

Following approval of the study protocol by the Ethics Committee of the City of Vienna (EK18-027-VK), a retrospective analysis of the patients’ information was carried out. Informed consent was waived due to the retrospective nature of the study.

TAVR procedures have already been described in detail [24]. The CM chosen during the TAVR procedure were: Iopamidol (300 mg of iodine/mL; 616 mOsmol/kg of water (Jopamiro, Bracco, Vienna, Austria))—a nonionic, low-osmolar monomer CM, and Iodixanol for patients with iodine intolerance or allergy (150 mg of iodine/mL; 290 mOsm/kg of water (Visipaque, GE Healthcare, Wädenswil, Switzerland))—a nonionic, iso-osmolar dimer [25,26].

Patients were diagnosed with a compromised renal function when their estimated glomerular filtration rate (eGFR) was <60 mL/min/1.73 m^2^, with eGFR being calculated using the Modification of Diet in Renal Disease (MDRD) equation [27]. Independent of left ventricular function, additional intravenous fluid therapy was administered in such patients in the form of 1000 mL of Ringer’s lactate solution over 3 h leading up to the procedure.

Blood samples were taken 24 h before the operation and then daily for 72 h following the operation. Procedural complications and AKI incidence were assessed in line with Valve Academic Research Consortium-2 criteria (VARC-2). AKI incidence was guided by the criteria’s cut-offs for changes in creatinine [28].

### 2.1. CM Volume Models

**Figure 1:**
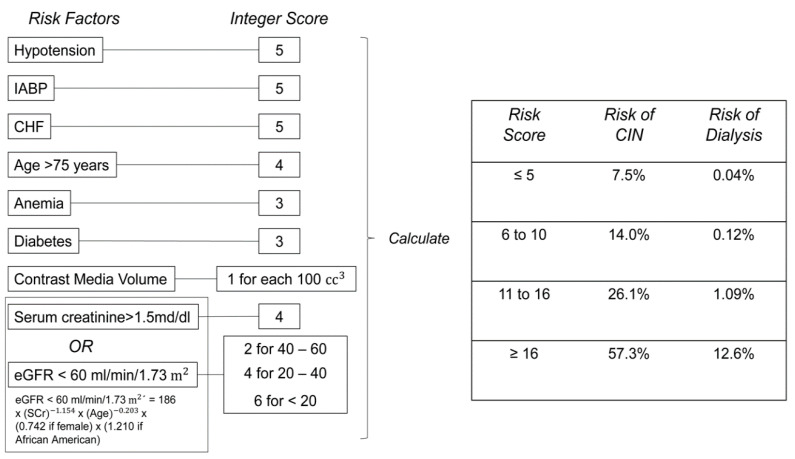
Risk Stratification Score Model designed by Mehran et al. to predict contrast induced nephropathy (CIN) following percutaneous coronary intervention (PCI) [7].

The risk of a post-procedural AKI based on CM dosage was calculated using our institutional and various previously published risk ratio models:

Heart Team Vienna: CM × SCr/BMI
Cigarroa et al.: (CM × Body Weight in kg)/SCr in mg per dL [6,29]
Mehran et al.: See Figure 1 [7]
Yamamoto et al.: CM × SCr/BW [8]

### 2.2. Statistical Analysis 

Based on the distribution of continuous variables, their data were either expressed as a median and interquartile range (IQR) or a mean and standard deviation (+/−SD) and they were compared using the Student’s *t*-test or the Mann–Whitney-U-test, respectively. Categorical data were expressed as absolute numbers and percentage, and compared with a Chi^2^-test or the Fisher’s exact test.

The study population was separated into two cohorts according to AKI occurrence. A univariate analysis was run with all patient variables measured and those whose odds ratio also yielded a *p*-value < 0.05 were included into a multivariate analysis. Survival rates for each cohort were calculated using Kaplan–Meier survival estimates and differences were compared using the log-rank test. CM dosage was compared between different AKI-stages (as defined by VARC-2) using the Kruskal–Wallis test (H-Test).

To examine the association between AKI and overall 1-year mortality, a Cox proportional hazards model was used to estimate hazard ratios and 95% confidence intervals (CI). Person-time was calculated from the date of the implantation to either death or the last available follow-up. The hazard ratio was stratified by the occurrence of postprocedural AKI and adjusted for baseline and procedural characteristics, including age, sex, body-mass index, dyslipidemia, hypertension, insulin-dependent diabetes, preprocedural renal impairment and serum creatinine levels, the EuroSCORE II, STS-score, and the left ventricular ejection fraction in a stepwise fashion.

Statistical analysis was completed using SPSS version 24.0 (IBM Corp, Armonk, NY, USA), and the reported *p*-values are 2-sided with an alpha level set at <0.05 for statistical significance.

## 3. Results

### 3.1. Baseline Characteristics

Of the 514 patients analyzed, 94 patients developed an AKI following the procedure of which 72 were stage one and 22 were stage three. The clinical characteristics of both the AKI and non-AKI cohort are presented in Table 1. Relative to the control group, the AKI cohort had a higher proportion of individuals suffering from dyslipidemia (67% vs. 56.9%; *p* = 0.026) and diabetes mellitus (24.5% vs. 14%; *p* = 0.012). Additionally, both the logistic EuroSCORE and the CHADS-VASC Score of AKI patients was significantly higher compared to their unaffected counterparts (21.6 ± 28.9 vs. 17.4 ± 19.6; *p* = 0.032 and 5.8 ± 1.4 vs. 5.2 ± 1.4; *p* = 0.014).

### 3.2. Procedural Characteristics

**Figure 2:**
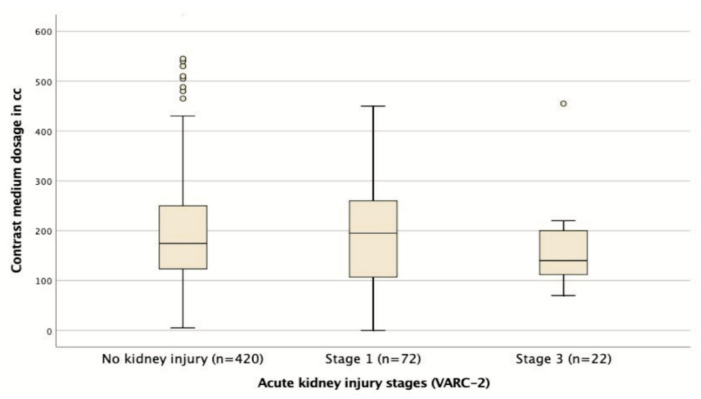
Average CM dosage administered to patients who developed different stages of AKI.

An overview of the procedural characteristics of patients is provided in Table 2. With respect to the secondary endpoints, the CM dose (AKI: 171 ± 136.3 vs. Non-AKI: 157 ± 127 mL; *p* = 0.968) was neither significantly different in all AKI patients nor between stage one and stage three AKI patients (*p* = 0.400; Figure 2). None of the models were found to be significantly different between the AKI and non-AKI patients, and the proportion of individuals exceeding the suggested cutoff level of 5 for the CM × SCr/BW ratio was in fact higher in the non-AKI cohort (AKI: 6.4% vs. Non-AKI: 13.6%; *p* = 0.030). Transfemoral-TAVR patients specifically had a higher incidence of AKI and received a larger CM dosage, but there was still no association between CM volume and AKI when transfemoral-TAVR and transapical-TAVR patients were analyzed separately.

AKI patients did spend more time in the intensive care unit (55.5 ± 141.5 vs. 21 ± 24 h; *p* < 0.001) and had a higher maximum creatinine level within the first 72 h following the procedure (2.5 ± 4.8 vs. 0.97 ± 0.3 mg/dL; *p* < 0.001). Furthermore, the number of packed red blood cell units (RBU) was higher in the AKI cohort (2.3 ± 4.3 vs. 0.9 ± 2.4 units; *p* = 0.004). AKI patients were also less likely to receive balloon-expanding valves (40.4% vs. 51.7%; *p* = 0.035) and accordingly required predilatation more often (72.3% vs. 61.2%; *p* = 0.042).

### 3.3. Adverse Events and Survival

**Figure 3:**
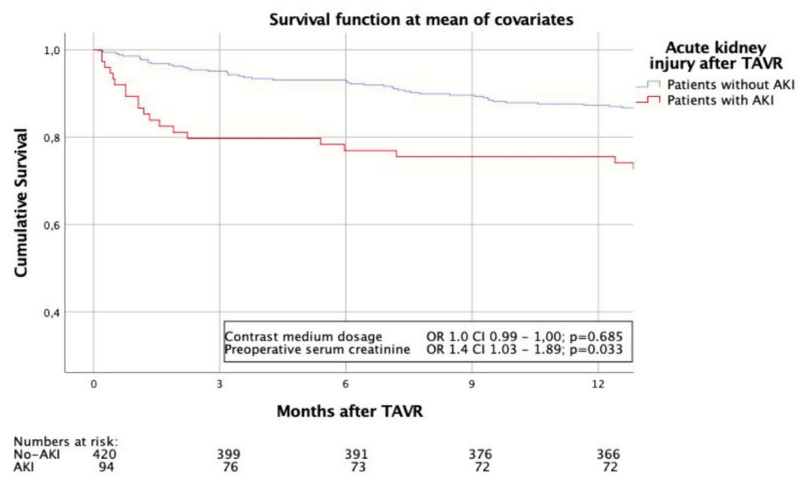
Survival of AKI and non-AKI patients at one year after adjusting for confounders.

Details regarding the post-interventional outcomes of the two cohorts following TAVR can be seen in Table 3. Major bleeding complication rates were almost double amongst AKI patients (13.8% vs. 7.4%; *p* = 0.027) in line with the higher rate of RBUs used in this cohort. 30-day mortality was significantly higher among AKI patients (8.5% vs. 1.4%; *p* = 0.001), and the number of people reaching the 30-day combined safety endpoint was correspondingly lower (56.4% vs. 88.3%; *p* < 0.001). Procedural success was significantly lower among AKI patients (77.7% vs. 90.7%; *p* = 0.006) and the need for reoperation was higher for both cardiac (13.8% vs. 7.6%; *p* = 0.035) and non-cardiac problems (13.8% vs. 6.4%; *p* = 0.013). After adjusting the Cox proportionate hazards model for covariates, a significant difference in 1-year survival between patients with and without postprocedural AKI was observed. While preprocedural serum creatinine levels were the only predictive factor in this model, the CM dosage had no influence on survival in this model (Figure 3).

### 3.4. Factors Associated with and Predictive of AKI

A logistic regression analysis was performed (Table 4). Following the univariate analysis, peripheral vascular disease, coronary vascular disease, prior PCI, left ventricular ejection fraction (LVEF), and systolic pulmonary artery pressure (sPAP) were found to be associated with AKI. Amongst the models that were assessed, only CM × SCr demonstrated a significant association with AKI. Upon conducting a multivariate analysis with all the aforementioned variables and each model, no independent predictive factor for AKI after TAVR was found.

## 4. Discussion

Our study shows that CM dosage is not a predictor of AKI even when analyzed using multiple different models. However, post-procedural AKI does affects both short- and longer-term mortality.

The link between CM dosage and renal dysfunction is documented in PCIs, but only a limited number of groups have been able to replicate this relationship for the TAVR procedure [5,7,8,14]. Yamamoto et al. recently trialed a model involving body weight, serum creatinine and CM volume, and found that a ratio over 2.7 was predictive of an AKI [8]. The present study showed no association between CM volume and AKI incidence though, a result that has been reported by other groups as well and reflects the conclusion of a meta-analysis which found no link between high CM volume and AKI after TAVR either [1,9,10,11,12,13,15,16,30].

The notion that the relationship between these two variables may be threshold dependent rather than continuous, was explored through our analysis of the data using several established risk ratio models. However, patients exceeding the threshold of five proposed by the Cigarroa risk ratio model for contrast-induced renal dysfunction were in fact more common amongst the non-AKI cohort [29]. Similarly, upon applying the risk stratification score proposed by Mehran et al. to our data, there was no significant relationship and the model was unable to differentiate the AKI and non-AKI cohort even after analyzing only patients who exceeded the high risk threshold of 10 suggested by the authors [7]. All of the aforementioned AKI risk ratio models were also neither significantly different between the AKI and non-AKI cohorts, nor did they not appear to be a predictive factor for AKI in our multivariate analysis.

The average CM volume used at our center is similar to those reported by other groups including Yamamoto et al.; therefore, it is unlikely to be the reason for the discrepancy [4,8,9]. Furthermore, despite the amount of CM administered varying radically among studies, this has not served as the divide between those who reported an association between CM volume and AKI and those who have not [1,2,8,9,10,11,12,13,14,15,16], suggesting that other factors may play a more prominent role in AKI development.

One school of thought that has emerged to explain analogous trends among patients undergoing contrast CT scans, is that a segment of the population will demonstrate renal decline that occurs irrespective of whether CM is administered or not [20]. In the context of intravenous CM administration, hypotension, nephrotoxic drugs, fluid restriction, and hemorrhage may contribute to CM-independent SCr increases that obscure the actual incidence of CM-induced nephropathy [19].

The TAVR procedure itself also involves several steps that have the potential to affect renal function. Endothelial damage triggered by intermittent periods of hypotension during rapid pacing could cause tubular ischemia by inhibiting the production of vasodilating substances such as nitric oxide. It has also been postulated that hypotension triggered by rapid pacing may cause renal hypoperfusion and therefore renal injury and AKI [31]. Although studies have reported no difference or association between utilization of rapid pacing and AKI incidence [32,33], there are conflicting reports with respect to the effects of the number of pacing runs/episodes on AKI development [15,34], and a recent study by Fefer et al. has shown that prolonged rapid pacing time increases the risk of AKI [34].

Advanced stages of atherosclerosis found in TAVR patients could affect AKI development too [10], especially as catheters dislodging cholesterol emboli which then travel to the kidneys and obstruct renal perfusion have been noted as a potential cause of renal damage in coronary angioplasty [35]; a comparable process may be occurring during catheter manipulation in TAVR given the higher incidence of dyslipidemia and diabetes mellitus found in our AKI patients. Advanced peripheral vascular disease is also thought to accentuate the adverse effects of RBU [2]. RBU transfusion is reported to be an independent predictor of AKI in TAVR and in a meta-analysis shown to be associated with AKI [8,33,36,37]. Accordingly, the number of RBU transfusions was significantly higher both amongst our patients and other reported cohorts [3,13,38]. The underlying pathophysiology for this association remains uncertain; it has been suggested that when RBCs are stored, their properties change which may adversely affect their oxygen transport. RBC structural changes have also been suggested to increase their viscosity and aggregability [39], which on a background of PVD may contribute to microembolic events that affect kidney function. Similarly, RBC membrane changes are thought to lead to hemolysis during storage which increases levels of free iron and hemoglobin in RBC transfusions, both of which are toxic to the kidney [40]. The notion that AKI is a consequence of these adverse outcomes rather their cause or the result of CM administration, is further merited by examining the characteristics of patients with fewer complications. Using patients discharged early as a proxy marker for an uncomplicated intervention, data showing that patients who left the hospital within two days of their TAVR procedure having lower rates of stage 2 or 3 AKIs but receiving the same CM volume as those discharged later, suggests it is procedural/post-procedural complications rather than CM volume that drives AKI [41].

Delineating the central players in AKI development is essential as our AKI patients had significantly higher 30-day mortality rates, fewer of them reached the 30-day combined safety endpoint and their 1-year mortality was significantly higher. The six-fold increase in 30-day mortality is close to the figures noted by others and the significantly higher logistic EuroSCORE in the AKI cohort has also been previously reported [13,42].

The biological plausibility of CM causing contrast associated nephropathy warrants precautionary measures such as hydration therapy. Current approaches to limiting CM dosage are appropriate, however additional restrictions on volume used should not come at the expense of withholding further pre-procedural assessment or sub-optimal procedural imaging in the interest of further reducing the risk of AKI, as other factors may be the primary drivers in AKI development after TAVR.

### Limitations

The study has several limitations including those inherent to a retrospective study. The significant difference in major bleeding complications observed between our AKI and non-AKI cohort may be a confounding variable in our analysis; considerable bleeding after TAVR may cause renal hypoperfusion and thus trigger a pre-renal AKI [43]. Rather than serving as a surrogate indicator for patients more likely to require blood transfusions and thus at risk of AKI, major vascular complications could be contributing the incidence of AKI and alter the data. The presence of other unidentified confounding variables cannot be excluded as well, as the cause of AKI is multifactorial. Indication bias stemming from doctors administering lower CM dosage to patients perceived as high-risk for AKIs may have also obscured the results; the almost identical pre-operative serum creatinine and proportion of individuals classified as renally impaired among the two cohorts however, indicates that this may have been well controlled. A lack of consensus regarding what fluids should be used for prehydration therapy to prevent contrast-associated nephropathy also means that our institution’s use of Ringers lactate was in accordance with recommendations by the American College of Radiology [44], but diverged from the guidelines set by European Society of Cardiology/European Association for Cardio-Thoracic Surgery [45]. A recent study in patients undergoing angioplasty or angiography though, showed similar incidences of contrast induced nephropathy (CIN) with either option suggesting that the choice in protocol is unlikely to have altered our findings [46]. Nevertheless, daily serum creatine measurements during the initiation phase of TAVR at our center were only performed during the first 72 h whereas the VARC-2 criteria suggest that the AKI diagnosis period should be extended up to 7 days [28]. This might have led to a potential underrepresentation of patients with late-onset AKI. The data from this study also stems from a single center and therefore the value in extrapolating its results may be limited.

## 5. Conclusions

The findings of the present analysis contradict the common misconception that acute kidney injury after TAVR is mainly caused by the contrast medium used during the procedure. As the underlying mechanisms remain unclear, future research needs to address different pathophysiological mechanisms and preventive measures as AKI negatively impacts short- and long-term survival.

## Figures and Tables

**Table 1:** Baseline clinical characteristics of AKI and non-AKI patients.

	Overall *n* = 514	AKI *n* = 94	Non-AKI *n* = 420	*p* Value
**Demographics**				
Age, mean (±SD)	81.3 (7.0)	82.9 (5.5)	81.0 (7.0)	0.385
Female, *n* (%)	323 (62.8)	61 (64.9)	262 (62.4)	0.370
Body mass index kg/m^2^, median (IQR)	25.9 (6.7)	26.7 (7.4)	24.7 (5.1)	0.369
**Risk Profile**				
EuroSCORE II, median (IQR)	4.8 (6.0)	6.6 (10.4)	5.4 (6.2)	0.103
Logistic EuroSCORE, median (IQR)	17.8 (20.4)	21.6 (28.9)	17.4 (19.6)	0.032
STS score, median (IQR)	4.5 (3.3)	5.8 (4.7)	4.6 (3.2)	0.288
Incremental risk score, median (IQR)	3 (8)	3 (9)	5 (11.5)	0.889
HAS-BLED score, median (IQR)	1 (1)	1 (1)	1 (1)	0.085
CHADS-VASC Score, mean (±SD)	5.3 (1.4)	5.8 (1.4)	5.2 (1.4)	0.014
**Chronic health Conditions and Risk Factors**				
Hypertension, *n* (%)	443 (86.2)	83 (88.3)	360 (85.7)	0.160
Dyslipidaemia, *n* (%)	302 (58.8)	63 (67)	239 (56.9)	0.026
Diabetes mellitus (IDDM), *n* (%)	82 (16.0)	23 (24.5)	59 (14)	0.012
COPD, *n* (%)	108 (21.0)	15 (16.0)	93 (22.1)	0.209
Peripheral vascular disease, *n* (%)	100 (19.5)	19 (20.2)	81 (19.3)	0.433
Cerebrovascular accident, *n* (%)	63 (12.3)	12 (12.8)	51 (12.1)	0.478
NYHA class III/IV, *n* (%)	442 (86.0)	79 (84.0)	363 (86.4)	0.908
Renal impairment eGFR < 60 mL/min/1.73 m², *n* (%)	87 (16.9)	16 (17.0)	71 (16.9)	0.891
eGFR mL/min/1.73 m², mean (±SD)	54.2 (25.6)	58.4 (25.6)	58.4 (26.7)	0.478
Creatinine mg/dL, median (IQR)	1.1 (0.6)	1.0 (0.4)	1.1 (0.6)	0.143
Creatinine clearance mL/min, mean (±SD)	48.7 (19.9)	52 (18.3)	51.5 (22.2)	0.841
Hematocrit %, median (IQR)	36.0 (6.7)	35.2 (6.0)	36.2 (6.9)	0.298
Dialysis, *n* (%)	1 (0.2)	0 (0)	1 (0.2)	0.820
Permanent pacemaker, *n* (%)	83 (16.1)	18 (19.1)	65 (15.5)	0.233
Prior myocardial infarction, *n* (%)	73 (14.2)	13 (13.8)	60 (14.3)	0.558
Prior PCI, *n* (%)	139 (27.0)	28 (29.8)	111 (26.4)	0.256
Previous CABG, *n* (%)	78 (15.2)	14 (14.9)	64 (15.2)	0.569
Previous valve surgery, *n* (%)	44 (8.6)	5 (5.3)	39 (9.3)	0.161
**Preoperative Echocardiographic Data**				
LVEF %, median (IQR)	55 (15)	55 (12.5)	55 (20)	0.565
Aortic valve area, mean (±SD)	0.7 (0.3)	0.8 (0.6)	0.7 (0.2)	0.368
Mean pressure gradient, mean (±SD)	46.7 (16.2)	49.8 (18.4)	46.3 (15.8)	0.154
Peak velocity m/sec, median (IQR)	4.2 (0.8)	4.2 (0.95)	4.19 (0.86)	0.875
sPAP, mean (±SD)	41.1 (20.7)	39 (23.1)	42.1 (20.1)	0.361

AKI—acute kidney injury; CABG—coronary artery bypass graft; CHA2DS2-VASc—congestive heart failure, hypertension, age >75 years, diabetes mellitus, stroke, or embolic event, vascular disease, age 65 to 74 years, sex; COPD—chronic obstructive pulmonary disease; eGFR—estimated glomerular filtration rate; EuroSCORE—European System for Cardiac Operative Risk Evaluation; IDDM—insulin dependent diabetes mellitus; IQR—interquartile range; HAS-BLED—hypertension, abnormal renal or liver function, elderly, stroke, prior major bleeding or predisposition, labile INR, drugs; Max.—maximum; LVEF—left ventricular ejection fraction; NYHA—New York Heart Association; PCI—percutaneous coronary intervention; SCr—serum creatinine; SD—standard deviation; sPAP—systolic pulmonary artery pressure; STS—Society of Thoracic Surgeons Predictive Risk of Mortality; TAVR—transcatheter aortic valve replacement.

**Table 2:** AKI risk models and procedural clinical characteristics of AKI and non-AKI patients.

	Overall *n* = 514	AKI *n* = 94	Non-AKI *n* = 420	*p* Value
**Contrast Medium Dose and AKI Risk Ratio Models**				
CM dose ml, median (IQR)	174.5 (131.3)	171 (136.3)	157 (127)	0.968
CM × SCr, median (IQR)	197.2 (163.2)	198.7 (155.3)	186.3 (166.5)	0.271
CM × SCr/BW ratio, median (IQR)	2.3 (2.7)	2.6 (2.7)	2.7 (2.3)	0.543
CM × SCr/BW ratio > 5, *n* (%)	63 (12.3)	6 (6.4)	57 (13.6)	0.030
CM × SCr/BMI ratio, median (IQR)	6.2 (7.8)	6.9 (6.8)	7.3 (6.8)	0.422
Risk Stratification Score Model, median (IQR)	12 (7)	12 (8)	12 (8)	0.918
Risk Stratification Score Model > 10, *n* (%)	337 (65.6)	63 (67)	274 (65.2)	0.370
**Procedural Variables**				
Transapical access, *n* (%)	256 (49.8)	42 (44.7)	214 (51)	0.174
Balloon expanding valve, *n* (%)	255 (49.6)	38 (40.4)	217 (51.7)	0.035
Predillatation necessary, *n* (%)	325 (63.2)	68 (72.3)	257 (61.2)	0.042
Postdillatation necessary, *n* (%)	59 (11.5)	8 (8.5)	51 (12.1)	0.188
Max. creatinine within 72 h mg/dL, median (IQR)	1.1 (0.6)	2.5 (4.8)	0.97 (0.3)	<0.001
Total hours in ICU, median (IQR)	21 (45)	55.5 (141.5)	21 (24)	<0.001
Total hours ventilated, median (IQR)	4 (7)	6 (17)	6 (4)	0.112
RBC units used, mean (±SD)	1.2 (2.7)	2.3 (4.3)	0.9 (2.4)	0.004
Any paravalvular leak, *n* (%)	229 (44.6)	38 (40.4)	191 (45.5)	0.149
Mean gradient post-implant, median (IQR)	9 (7)	4.5 (9.8)	6 (11)	0.244
Max. gradient post-implant, median (IQR)	17 (15)	8 (16)	16 (18.5)	0.956
Max. flow post-implant, mean (±SD)	2.1 (1)	2 (0)	2.1 (1)	0.347

BMI—body mass index; BW—body weight; CM—contrast medium; ICU—intensive care unit; RBU—red blood cell; other abbreviations as in Table 1.

**Table 3:** Adverse events of AKI and non-AKI patients.

	Overall *n* = 514	AKI *n* = 94	Non-AKI *n* = 420	*p* Value
Myocardial infarction, *n* (%)	2(0.4)	1 (1.1)	1 (0.2)	0.321
Neurological adverse event, *n* (%)	12 (2.3)	2 (2.1)	10 (2.4)	0.619
Major vascular complication, *n* (%)	9 (1.8)	2 (2.1)	7 (1.7)	0.722
Major bleeding complication, *n* (%)	44 (8.6)	13 (13.8)	31 (7.4)	0.027
New AV-block, *n* (%)	59 (11.5)	6 (6.4)	53 (12.6)	0.121
New bundle branch block, *n* (%)	78 (15.2)	16 (17.0)	62 (14.8)	0.372
New atrial fibrillation, *n* (%)	53 (10.3)	8 (8.5)	45 (10.7)	0.388
New pacemaker implanted, *n* (%)	70 (13.6)	16 (17.0)	54 (12.9)	0.129
Reoperation for valvular dysfunction, *n* (%)	4 (0.8)	1 (1.1)	3 (0.7)	0.540
Reoperation for bleeding/tamponade, *n* (%)	14 (2.7)	4 (4.3)	10 (2.4)	0.181
Reoperation for other cardiac problems, *n* (%)	45 (8.8)	13 (13.8)	32 (7.6)	0.035
Reoperation for non-cardiac problems, *n* (%)	40 (7.8)	13 (13.8)	27 (6.4)	0.013
Conversion to open surgery, *n* (%)	4 (0.8)	1 (1.1)	3 (0.7)	0.535
Unplanned valve-in-valve implantation, *n* (%)	8 (1.6)	2 (2.1)	6 (1.4)	0.418
Length of stay after TAVR in days, median (IQR)	10 (8)	15.5 (14.5)	12 (8)	0.150
Procedural success, *n* (%)	454 (88.3)	73 (77.7)	381 (90.7)	0.006
30-day combined safety endpoint, *n* (%)	424 (82.5)	53 (56.4)	371 (88.3)	<0.001
30-day all-cause mortality, *n* (%)	14 (2.7)	8 (8.5)	6 (1.4)	0.001
1-year all-cause mortality, *n* (%)	77 (15.0)	22 (23.4)	55 (13.1)	0.007

AV—atrioventricular; other abbreviations as in Table 1 and Table 2.

**Table 4:** Univariate and multivariate analysis of predictive factor for AKI.

	Univariate Analysis			Multivariate Analysis		
	OR	95% CI	*p* Value	OR	95% CI	*p* Value
Peripheral Vascular Disease	0.04	0.004–0.437	0.008	0.01	−0.096–0.099	0.995
Coronary Vascular Disease	5.51	1.020–29.753	0.047	0.64	−0.033–0.161	0.172
Prior PCI	0.12	0.019–0.734	0.022	−0.79	−0.169–0.011	0.100
LVEF %	1.09	1.010–1.184	0.027	0.00	−0.003–0.004	0.983
sPAP	0.97	0.934–0.996	0.030	0.00	−0.002–0.002	0.643
CM x SCr	0.97	0.953–0.991	0.004	0.00	0.000–0.001	0.134
CM × SCr/BW ratio	0.77	0.349–1.707	0.523			
CM × SCr/BMI ratio	1.50	0.922–2.454	0.102			
Risk Stratification Score Model	1.10	0.990–1.216	0.077			
CM × SCr/BW ratio > 5	0.78	0.200–3.059	0.724			
Risk Stratification Score Model > 10	1.03	0.438–2.436	0.940			

CI—confidence interval; OR—odds ratio; other abbreviations as in Table 1, Table 2 and Table 3.
